# Biochemical and Morphological Changes Triggered by Nitrogen Stress in the Oleaginous Microalga *Chlorella vulgaris*

**DOI:** 10.3390/microorganisms10030566

**Published:** 2022-03-05

**Authors:** Tan Liu, Zhihui Chen, Yunhua Xiao, Mingmin Yuan, Chenkai Zhou, Gang Liu, Jun Fang, Bo Yang

**Affiliations:** Hunan Provincial Engineering Research Center of Applied Microbial Resources Development for Livestock and Poultry, College of Bioscience and Biotechnology, Hunan Agricultural University, Changsha 410125, China; liutan@stu.hunau.edu.cn (T.L.); chenzhihui@stu.hunau.edu.cn (Z.C.); huazipiaoling.123@163.com (Y.X.); yuanmingmin@stu.hunau.edu.cn (M.Y.); zhouchenkai@stu.hunau.edu.cn (C.Z.); gangle.liu@gmail.com (G.L.)

**Keywords:** microalgae, nitrogen limitation, nitrogen starvation, biomass, lipid, *Chlorella vulgaris*

## Abstract

Oleaginous microalgae have been considered promising sources of biodiesel due to their high lipid content. Nitrogen limitation/starvation is one of the most prominent strategies to induce lipid accumulation in microalgae. Nonetheless, despite numerous studies, the mechanism underlying this approach is not well understood. The aim of this study was to investigate the effect of nitrogen limitation and starvation on biochemical and morphological changes in the microalga *Chlorella vulgaris* FACHB-1068, thereby obtaining the optimal nitrogen stress strategy for maximizing the lipid productivity of microalgal biomass. The results showed that nitrogen limitation (nitrate concentration < 21.66 mg/L) and starvation enhanced the lipid content but generally decreased the biomass productivity, pigment concentration, and protein content in algal cells. Comparatively, 3-day nitrogen starvation was found to be a more suitable strategy to produce lipid-rich biomass. It resulted in an increased biomass production and satisfactory lipid content of 266 mg/L and 31.33%, respectively. Besides, nitrogen starvation caused significant changes in cell morphology, with an increase in numbers and total size of lipid droplets and starch granules. Under nitrogen starvation, saturated fatty acids (C-16:0, C-20:0, and C-18:0) accounted for the majority of the total fatty acids (~80%), making *C. vulgaris* FACHB-1068 a potential feedstock for biodiesel production. Our work may contribute to a better understanding of the biochemical and morphological changes in microalgae under nitrogen stress. Besides, our work may provide valuable information on increasing the lipid productivity of oleaginous microalgae by regulating nitrogen supply.

## 1. Introduction

Fossil-fuel depletion, greenhouse effects, and global warming have led to the current energy crisis worldwide [1]. This has prompted efforts to develop renewable and sustainable energy sources. In recent years, biodiesel has generally been recognized as one of the most reliable renewable energy sources and has been receiving increasing attention. Biodiesel can serve as a promising alternative biofuel due to its eco-friendly characteristics such as no net increased release of carbon dioxide and aromatic compounds, while having very similar functional properties to fossil fuels [2,3]. Biodiesel is usually produced from raw materials such as plant oils or animal fats. Nonetheless, the availability of these raw materials does not meet the requirements of the potential consumer market for biodiesel [4]. Thus, recent research initiatives have established that microalgae biomass has emerged as a potential raw material for biodiesel production [3].

Microalgae are sunlight-driven green cell factories that can fix atmospheric carbon dioxide and convert it to different value-added biomolecules such as proteins, lipids, carbohydrates, and carotenoids [5]. As compared to other raw materials (e.g., crops and animal fat), microalgae have distinct desirable advantages for use as a biodiesel feedstock, such as simple growth requirements, high photosynthetic efficiency, rapid growth, and high lipid content [6,7]. Many microalgae have good adaptability to various ecological environments. They have the ability to grow in various sources of water such as freshwater, saline water, or wastewater. In particular, they are capable of using wastewater as a source of nutrients for growth and thus can be used for wastewater treatment. Microalgae-based wastewater treatment can reduce the energy use of waste management strategies and regenerate nutrients derived from waste effluents such as nitrogen and phosphorus [8,9]. In addition, microalgae can produce lipids with simultaneous production of other fine valuable bioactive compounds such as proteins and carbohydrates [10,11], thereby offsetting the lipid production cost and enhancing their economic feasibility as a source of biodiesel [12,13]. Moreover, microalgae are capable of capturing carbon dioxide in the atmosphere and flue gas in the form of photosynthesis under controlled conditions. This carbon bio-mitigation process meets the requirements of low-carbon development strategy advocated by many countries such as China [14,15,16]. Hence, microalgae can be used as an essential source for third-generation biodiesel production.

In order to enhance the economic feasibility of microalgae culture for biodiesel production, the productivities concerning biomass and lipid serve as vital criteria to be optimized. Reports have shown that the biomass productivity and biochemical composition (e.g., lipid content) of microalgae can be easily changed by adjusting the medium nutrients or physical cultivation conditions to obtain a desirable yield [17]. Various nutritional strategies such as nitrogen, phosphorus, iron, and silicon deficiency and various physical strategies such as temperature and light intensity have been investigated to enhance the lipid content in algal cells [18]. Among them, nitrogen limitation or starvation is generally deemed as the most effective stimulator to trigger lipid accumulation in microalgae [19]. However, in spite of numerous studies, the changes in cell growth, morphology, and biochemical composition of microalgae when subjected to nitrogen limitation or starvation are not yet well understood. Nitrogen plays an important role in facilitating cell growth. Microalgae are capable of using nitrogen as an essential component to build proteins, chlorophylls, and nucleic acids, among other molecules [20]. 

Lipid productivity is the product of both lipid content and biomass productivity [18]. In many cases, enhanced lipid content in response to nitrogen starvation is negatively correlated with lipid productivity because of the lower biomass productivity [18,21]. This disadvantage can be overcome by using intermediate nitrogen stress or a two-stage nitrogen starvation strategy [21,22]. Intermediate nitrogen concentration was found to give rise to higher biomass and lipid productivities [22]. The two-stage nitrogen starvation strategy has elicited expanding interests recently, in which nitrogen-sufficient feed was first employed to efficiently produce biomass, followed by nitrogen-free feed to enhance the lipid content [21].

Among the oleaginous microalgae, *Chlorella vulgaris* has elicited considerable attention. It is widely used as a promising alternative source of lipids for use in biodiesel production due to its rapid growth, high lipid content, strong adaptability, and low susceptibility to bacterial contamination [23]. Besides, *C. vulgaris* has a solid research basis for the production of bioactive compounds and bioenergy from laboratory to large scale. This will greatly facilitate the commercial use of *C. vulgaris*-based biodiesel production [24]. Reports have shown that the intracellular biochemical compositions and morphological changes in *C. vulgaris* are mainly affected by changes in nutritional conditions, light intensity, carbon dioxide concentration, and air flow rate [25,26,27,28,29]. 

The aim of the present study was to investigate the effects of nitrogen limitation and sequential nitrogen starvation on biochemical and morphological changes in the oleaginous microalga *C. vulgaris* FACHB-1068. It aimed to determine which nitrogen stress strategy is more useful to maximize the biomass and lipid production of this algal strain. Biochemical and morphological data including dry cell weight, biomass productivity, photosynthetic pigment concentrations, lipids, proteins, carbohydrates, and fatty acid profile were analyzed for a better understanding of lipid accumulation mechanisms under nitrogen stress in microalgae. These results may also provide valuable information on increasing the lipid productivity of oleaginous microalgae by regulating nitrogen supply.

## 2. Materials and Methods

### 2.1. Strain and Cultivation Conditions

The microalgal strain *C. vulgaris* FACHB-1068 used in this study was obtained from the Freshwater Algae Culture Collection at the Institute of Hydrobiology (FACHB). This strain was cultured and maintained in Basal medium [30], which contained (per liter) 1250 mg KNO_3_, 1250 mg KH_2_PO_4_, 1000 mg MgSO_4_·_7_H_2_O, 500 mg Na_2_EDTA·2H_2_O, 114.2 mg H_3_BO_3_, 111 mg CaCl_2_·2H_2_O, 49.8 mg FeSO_4_·7H_2_O, 88.2 mg ZnSO_4_·7H_2_O, 14.2 mg MnCl_2_·4H_2_O, 7.1 mg MnO_3_, 15.7 mg CuSO_4_·5H_2_O, and 4.9 mg Co(NO_3_)_2_·6H_2_O. The pH value of the medium was adjusted to 6.1 prior to autoclaving. Algal cells were passaged once a month by inoculating them to liquid medium at 10% (*v*/*v*). For the nitrogen limitation experiments, *C. vulgaris* was cultivated in Basal medium with various initial nitrate concentrations of 173.25, 86.63, 43.31, 21.66, 10.83, and 0 mg/L. As KNO_3_ (1250 mg/L) was used as the only nitrate source in the Basal medium, the initial nitrate concentration in the Basal medium can be calculated as 173.25 mg/L. The nitrate (KNO_3_) solution with the concentration of 173.25 mg/L was prepared as the mother liquor. The other nitrate concentrations of 86.63, 43.31, 21.66, and 10.83 mg/L were thus prepared by gradient dilution. For the sequential nitrogen starvation experiments, this process was divided into two stages. In the first stage, algal cells were first cultured in Basal medium with full nitrate concentration (i.e., 173.25 mg/L) for 12, 6, and 3 days. In the second stage, microalgal cells grown in full nitrate medium with different incubation times were collected by centrifugation at 6000 rpm for 15 min, washed twice with distilled water, and then transferred to new nitrogen-free medium and grown for 3, 6, and 9 days. The nitrogen-free medium was the same as the Basal medium except without the addition of KNO_3_. Algal culture grown in full nitrate medium (i.e., 173.25 mg/L) for 15 days was used as a control. In all cases, 200 mL of liquid medium in a 500 mL Erlenmeyer flask was inoculated at 10% (*v*/*v*). The cultures were incubated at 25 ± 1 °C in a light incubator under continuous illumination of 80 μmol photons m^−2^ s^−1^ under a photoperiod of 12:12 h light/dark cycle. The photon flux density was measured by a light meter (TES-1330A, Taiwan, China) in the center of flask vertical to rays of light. The flasks were manually shaken three times a day.

### 2.2. Determination of Dry Cell Weight and Pigments

Biomass was harvested by centrifugation at 12,000 rpm for 10 min. The pellet was washed twice with distilled water, and the dry cell weight was determined after drying to constant weight in the oven at 80 °C.

Chlorophyll *a* and carotenoids in harvested cells were extracted with 1 mL of methanol for 5 h at 4 °C. The extract was then centrifuged, and an ultraviolet–near-infrared spectrophotometer UV-3600 Plus (Shimadzu, Japan) was used to determine the absorbance of the supernatant at 480, 652, 665, and 750 nm. The pigment concentrations were calculated by the following equations [31]:

[Chlorophyll *a*] mg/L = 16.5169 × A_665_ − 8.0962 × A_652_

[Carotenoid] mg/L = 4 × A_480_

The absorbance at 652, 665, and 480 nm was corrected by subtracting the absorbance at 750 nm. 

### 2.3. Determination of Residual Nitrate Concentration

The residual nitrate concentration was determined using the concentrated sulfuric acid–salicylic acid method [32]. Briefly, samples were centrifuged at 12,000 rpm for 10 min. The supernatant was collected and diluted. Four hundred microliters of salicylic acid was added to 0.1 mL of diluent, and the mixture was incubated at room temperature for 20 min. After the addition of 9.5 mL of 2 M sodium hydroxide and cooling to room temperature, the absorbance was measured spectrophotometrically at 410 nm. The nitrate concentration was determined from the standard curve. 

### 2.4. Electron Microscopy Analysis

Algal cells were sedimented by centrifugation at 12,000 rpm for 10 min. The cell pellets were fixed with 2.5% glutaraldehyde. After fixation for 24 h, samples were rinsed three times with 0.1 M phosphate buffer (pH 7.0) and then fixed with 1 % osmic acid for 1–2 h at 4 °C. The samples were dehydrated by gradient concentration of ethanol and finally embedded with an embedding agent. Ultrathin sections were cut with an ultramicrotome (Leica EM UC 7; Leica-Microsystems, Vienna, Austria). The microphotographs were captured using an H-7650 transmission electron microscope (Hitachi, Tokyo, Japan) at 80 kV.

### 2.5. Lipid and Fatty Acid Analysis

Total lipids were extracted from lyophilized cells by gravimetric analysis [33]. Briefly, 50 mg of lyophilized algal powder was finely ground in a mortar and was extracted in a mixture of chloroform, methanol, and water with a volume ratio of 2:1:0.75. The chloroform layer was collected into a preweighted 10 mL centrifuge tube. After drying with nitrogen gas, the centrifuge tube with residuals was dried in a dry oven at 80 °C until constant weight and weighed.

Fatty acids were analyzed according to the method described by Bigogno et al. [34]. Twenty milligrams of lyophilized algal powder was incubated at 50 °C for 10 h in a solvent mixture consisting of 1 mL of toluene and 2 mL of 1% methanol sulfate (*v*/*v*). Heptadecanoic acid was used as the internal standard. Fatty acids from lipids were converted to fatty acid methyl esters (FAMEs). After extracting with hexane, FAMEs were analyzed by QP 2010 SE gas chromatograph–mass spectrometer (Shimadzu, Kyoto, Japan) using a Stabilwax-DA capillary column (30 m × 0.25 mm × 0.25 μm) (Shimadzu, Japan). The program used increased the temperature from 150 to 200 °C at a rate of 10 °C per min, and then to 220 °C at 6 °C per min, followed by a hold at 220 °C for 10 min. FAMEs were identified by comparing their fragmentation patterns with those in the NIST library. 

### 2.6. Determination of Carbohydrate and Protein Content 

For carbohydrate content, 10 mg of lyophilized algal powder was extracted with 5 mL of distilled water in a boiling water bath for 30 min, and then this process was repeated three times. The extracts were collected together and centrifuged, and the supernatant was transferred into a 25 mL volumetric flask and diluted to volume with distilled water. The carbohydrate content was determined using phenol–sulfuric acid method [35]. 

For protein content, 200 μL of 1 M sodium hydroxide was added to 10 mg of lyophilized algal powder and hydrolyzed in a water bath at 80 °C for 10 min. Then, 1.8 mL of distilled water was added to the hydrolysate, and the mixture was centrifuged at 12,000 rpm for 30 min. The supernatant was collected in a new centrifuge tube. Each sample was extracted twice and the supernatants were collected together [36]. Finally, the protein content was measured using a protein analysis kit (Bradford P0006; Beyotime, Shanghai, China).

### 2.7. Statistical Analysis

All experiments were performed in triplicate, and the data were expressed as mean value ± standard deviation (SD). SPSS 25.0 software package (Chicago, IL, USA) was used for statistical analysis. Data were analyzed using one-way analysis of variance (ANOVA) and *p* < 0.05 was considered statistically significant.

## 3. Results and Discussion

### 3.1. Effect of Nitrogen Limitation and Nitrogen Starvation on Cell Growth

Nitrogen is a major component in many biomolecules such as proteins, chlorophylls, and nucleic acids, which are important in sustaining algal growth [19]. Nitrogen limitation or starvation often resulted in an increase in lipid and carbohydrate content but at the expense of biomass productivity [37]. However, due to this tradeoff and in order to increase the economic production of lipid-rich algal biomass, it is necessary to investigate the optimal nitrogen concentration and nitrogen starvation periods. In our studies, we conducted two experiments. In the first nitrogen limitation experiment, *C. vulgaris* FACHB-1068 was subjected to various concentrations of nitrate ranging from 173.25 to 0 mg/L. This aims at exploring the optimal nitrate concentration for the production of higher lipid and biomass. In the second nitrogen starvation experiment, algal cells were first cultured in full nitrate medium for 12, 6, and 3 days and then transferred to nitrate-free medium and grown for 15 days in total. This aims at finding out the optimal nitrogen starvation period for maximizing the biomass and lipid production in *C. vulgaris* FACHB-1068.

**Figure 1:**
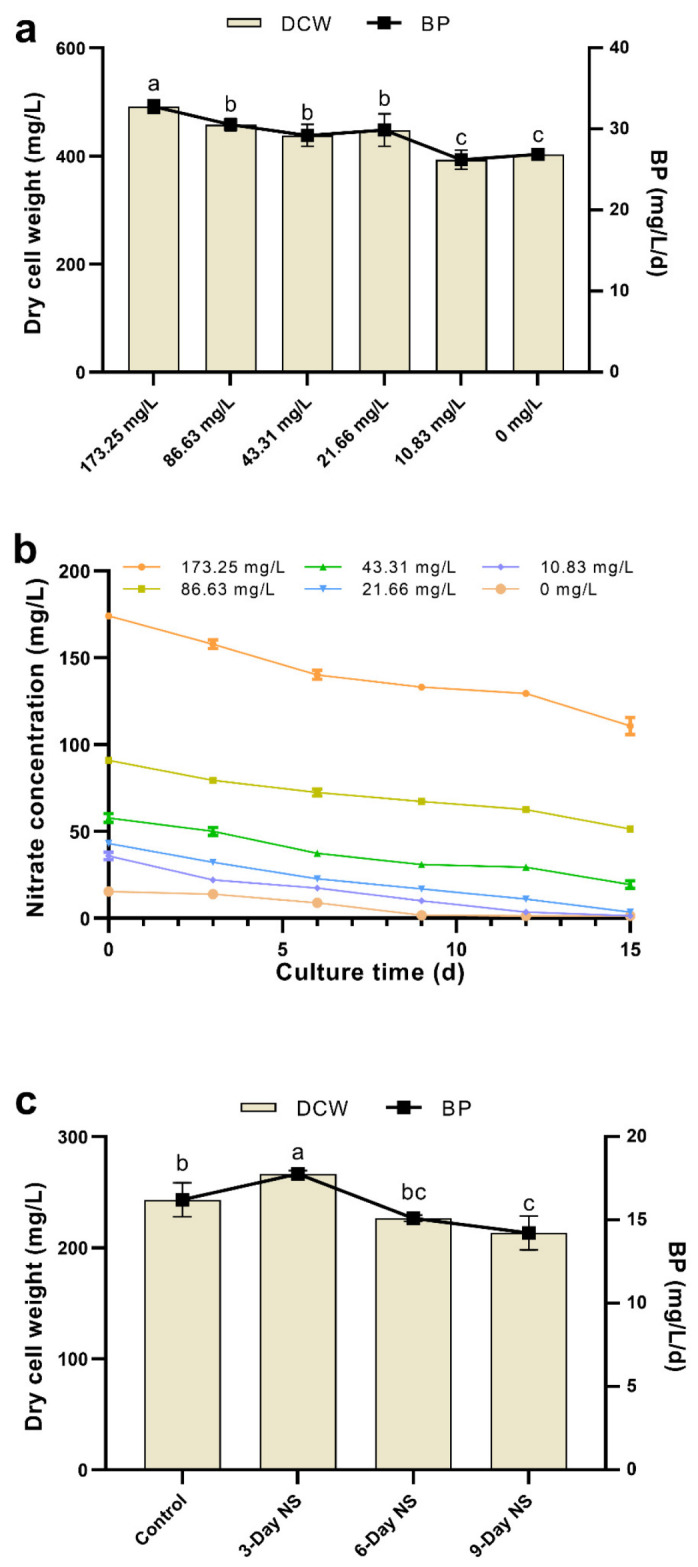
(**a**) Effect of nitrogen limitation on DCW and biomass productivity of *C. vulgaris* FACHB-1068. (**b**) Nitrate consumption curve of *C. vulgaris* FACHB-1068 under different nitrate concentrations ranging from 173.25 to 0 mg/L. (**c**) Effect of different nitrogen starvation conditions on DCW and biomass productivity of *C. vulgaris* FACHB-1068. Control, algal culture grown in nitrate-sufficient medium for 15 days; 3-Day NS, 6-Day NS, and 9-Day NS, algal cultures grown in nitrate-sufficient medium for 12, 6, and 3 days, respectively, followed by nitrogen starvation for 3, 6, and 9 days, respectively. Error bars represent SD (*n* = 3). Values with different letters represent significant difference (*p* < 0.05) between treatments.

For the nitrogen limitation experiment, Figure 1a shows the effect of nitrate limitation on dry cell weight (DCW) and biomass productivity (BP) of *C. vulgaris* FACHB-1068. As the nitrate concentration decreased from 173.25 to 0 mg/L, the DCW slightly decreased. This might be explained by nitrogen limitation slowing down the metabolism and cell proliferation of *C. vulgaris*, which was similar to the results observed in *Scenedesmus* sp., *C. minutissima*, and *Picocystis salinarum* [17,38]. However, the DCW was not significantly affected by nitrogen limitation when the nitrate concentration was above 10.83 mg/L (*p* > 0.05, Figure 1a). This indicated that *C. vulgaris* FACHB-1068 could grow in a culture containing 87.5% less nitrate (21.66 mg/L) compared to Basal medium (173.25 mg/L), which could enhance the economic feasibility of cultivation. The relatively low DCWs of 393.33 ± 17.56 mg/L and 403.33 ± 7.64 mg/L were obtained in cultures containing 10.83 and 0 mg/L nitrate, respectively (Figure 1a), which are 20.00% and 17.97% lower compared to the culture containing 173.25 mg/L nitrate. BP was also not significantly affected by nitrogen limitation when the nitrate concentration was above 10.83 mg/L (*p* > 0.05, Figure 1a). In the culture containing 10.83 mg/L nitrate, BP gradually decreased to only 26.22 ± 1.17 mg/L/day.

In our preliminary experiments of nitrogen limitation, we found that most of the nitrate still remained in the Basal medium (173.25 mg/L nitrate) after 15 days of culture. During the cultivation period, *C. vulgaris* FACHB-1068 consumed only 57–67 mg/L of nitrate, indicating that ~62% of the initial nitrate still remained in the medium at the end of cultivation. This was uneconomic undoubtedly. Accordingly, in order to determine the appropriate nitrate concentration for cell growth, we designed the nitrate consumption experiment. Algal cells were grown for 15 days in cultures containing the initial nitrate concentration ranging from 173.25 to 0 mg/L and were sampled every three days to determine the nitrate consumption. It was found that the trend of nitrate consumption was similar and decreased over time in cultures with various starting nitrate concentrations (Figure 1b). This was similar to the results obtained in previous studies [39]. In cultures containing 173.25, 86.63, and 43.31 mg/L nitrate, there was still more than ~45% nitrate left in the medium at the end of cultivation. This implied that algal growth might be affected by other factors (such as phosphorus concentration, light intensity, temperature, and pH) besides the starting nitrate concentration. In contrast, *C. vulgaris* FACHB-1068 almost exhausted the nitrate in cultures containing below 43.31 mg/L nitrate.

For the nitrogen starvation experiment, Figure 1c shows the DCW and BP of *C. vulgaris* FACHB-1068 under sequential nitrogen starvation. Three-day nitrogen starvation resulted in an increase in DCW (266.67 ± 2.89 mg/L) as compared to the control (243.33 ± 15.28 mg/L). This might be due to the algal strain grown in full nitrate medium for a long period of 12 days probably enabling itself to enter the stationary phase. Although it was subsequently subjected to 3-day nitrogen starvation, the redistribution of intracellular nitrogen pools such as chlorophylls and proteins supported the cell growth under nitrogen-depleted conditions for the first few days [22]. However, 6-day and 9-day nitrogen starvation showed a decrease in DCW as compared to the control (Figure 1c). This could be explained by the relatively short culture period in full nitrate medium and relatively long culture period in nitrogen-free medium probably leading to low cell numbers and a resultant decreased DCW. Compared to nitrogen limitation, nitrogen starvation also had a similar effect on BP. BP was found to increase under 3-day nitrogen starvation but decrease under both 6-day and 9-day nitrogen starvation (Figure 1c). These results suggested that a two-stage nitrogen starvation might be an effective strategy to sustain enough biomass for possible lipid production.

### 3.2. Effect of Nitrogen Limitation and Starvation on Pigment Concentration

**Figure 2:**
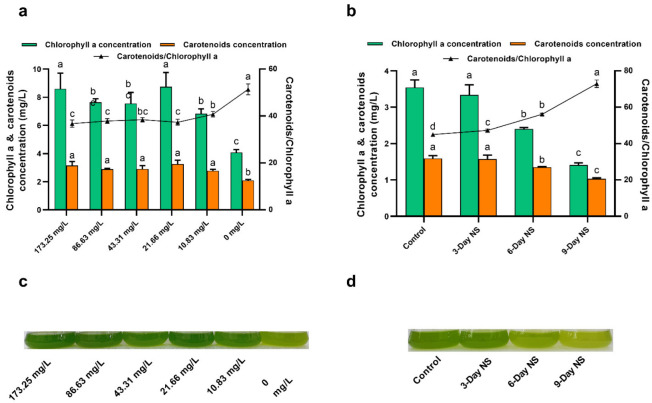
(**a**) Effect of nitrogen limitation on chlorophyll *a* and carotenoid concentrations of *C. vulgaris* FACHB-1068. (**b**) Effect of nitrogen starvation on chlorophyll *a* and carotenoid concentrations of *C. vulgaris* FACHB-1068. (**c**) Culture flasks of *C. vulgaris* FACHB-1068 under nitrogen limitation conditions. (**d**) Culture flasks of *C. vulgaris* FACHB-1068 under nitrogen starvation conditions. Control, algal culture grown in nitrate-sufficient medium for 15 days; 3-Day NS, 6-Day NS, and 9-Day NS, algal culture grown in nitrate-sufficient medium for 12, 6, and 3 days, respectively, followed by nitrogen starvation for 3, 6, and 9 days, respectively. Error bars represent SD (*n* = 3). Values with different letters represent significant difference (*p* < 0.05) between treatments.

Nitrogen limitation or starvation can not only boost lipid production, but also lead to several metabolic responses, including the degradation of nitrogen-containing bio-compounds such as protein and chlorophyll and the degradation of chloroplasts and thus a reduction in photosynthetic efficiency [19]. Chlorophyll *a* is of great importance for light harvesting in photosystems and represents the principal pigment in photosynthetic microalgae. Carotenoids, particularly secondary carotenoids, play a major role in protecting algal cells from oxidative stresses caused by excess light [36]. Therefore, we determined the concentration of photosynthetic pigments in order to evaluate the effect of nitrogen limitation and starvation on photosynthesis. As seen from Figure 2a, both chlorophyll *a* and carotenoid concentrations were almost not greatly affected under the nitrate concentration of above 10.83 mg/L. In other words, there was little difference in pigment concentrations under higher nitrate concentrations (>10.83 mg/L). In contrast, cultures containing lower nitrate concentrations (10.83 and 0 mg/L) had obviously lower pigment concentrations. These results were in accordance with the DCW data mentioned above (Figure 1a). The DCW decreased greatly under lower nitrate concentrations compared to control (173.25 mg/L nitrate), which can be explained by the lower pigment concentration. Compared to control (173.25 mg/L nitrate), a 52.56% reduction in chlorophyll *a* concentration and a 33.65% reduction in carotenoid concentration were observed in nitrogen-free culture (0 mg/L nitrate). This could also be seen in the culture flask coloration, with intense green pigmentation under nitrate concentrations above 10.83 mg/L, light green pigmentation under 10.83 mg/L nitrate, and yellowish pigmentation at 0 mg/L nitrate (Figure 2c). However, the ratio of carotenoids/chlorophyll *a* was found to almost increase from 36.78% to 51.40% as nitrate concentration decreased from 173.25 to 0 mg/L. In particular, there was an obvious increase in the ratio of carotenoids/chlorophyll *a* under lower nitrate concentrations (10.83 and 0 mg/L, Figure 2a). These results suggested a possible decrease in light-harvesting complex and PSII activity. Reports have shown that higher carotenoid levels and carotenoids/chlorophyll ratio under stress conditions may play a vital role in protecting algal cells from oxidative stresses caused by excess light [36,40].

For the nitrogen starvation experiment, similarly, both chlorophyll *a* and carotenoid concentrations decreased as the period of nitrogen starvation increased from 0 to 9 days (Figure 2b). Compared to control, except 3-day nitrogen starvation, 6-day and 9-day nitrogen starvation significant decreased pigment concentrations (*p* < 0.05). This also can be corroborated by culture flask coloration, with green pigmentation for control and 3-day nitrogen starvation, light green pigmentation for 6-day nitrogen starvation, and yellowish pigmentation for 9-day nitrogen starvation (Figure 2d). Notably, the chlorophyll *a* concentration was slightly reduced under 3-day nitrogen starvation as compared to control (Figure 2b). As mentioned above, 3-day nitrogen starvation resulted in an increase in both DCW and BP compared to control (Figure 1a). These observations possibly suggested that the nitrogen-containing chlorophyll *a* might have been degraded for reutilization of the nitrogen so as to support further cell growth when the nitrogen in the medium was depleted for 3 days [22]. The ratio of carotenoids/chlorophyll *a* also increased from 44.99 ± 0.58% to 72.89 ± 2.09% as the period of nitrogen starvation increased from 0 to 9 days (Figure 2b). In addition, as shown in Figure 2, sequential nitrogen starvation seems to have a greater effect on pigment concentrations than nitrogen limitation for *C. vulgaris* FACHB-1068.

### 3.3. Effects of Nitrogen Limitation and Starvation on Biochemical Composition

**Figure 3:**
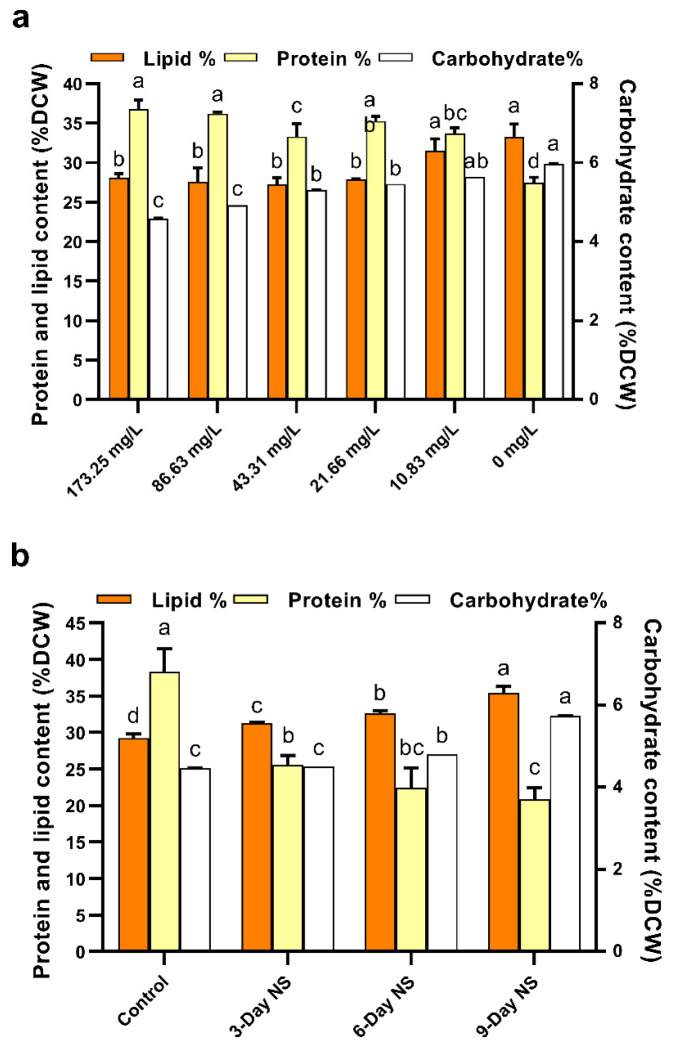
(**a**) Effects of nitrogen limitation on carbohydrate, protein, and total lipid contents of *C. vulgaris* FACHB-1068. (**b**) Effects of different nitrogen starvation durations on total lipid, carbohydrate, and protein contents in Chlorella vulgaris FACHB-1068. Control, algal culture grown in nitrate-sufficient medium for 15 days; 3-Day NS, 6-Day NS, and 9-Day NS, algal culture grown in nitrate-sufficient medium for 12, 6, and 3 days, respectively, followed by nitrogen starvation for 3, 6, and 9 days, respectively. Error bars represent SD (*n* = 3). Values with different letters represent significant difference (*p* < 0.05) between treatments.

Nitrogen limitation or starvation affects carbon distribution in microalgae. Carbon fixed by photosynthetic microalgae is mainly used to produce intracellular biomolecules such as proteins, lipids, and carbohydrates. Therefore, carbon distribution in microalgae can be reflected by changes in the biochemical composition of algal cells [19]. The biochemical composition of *C. vulgaris* FACHB-1068 in response to nitrogen limitation and starvation is presented in Figure 3. Because nitrogen is necessary for protein synthesis, nitrogen deficiency has an impact on protein concentrations and thus inhibits cell division [38]. 

As presented in Figure 3a, there was little difference in protein content of *C. vulgaris* FACHB-1068 under higher nitrate concentrations (>10.83 mg/L). However, the protein content generally decreased from 36.83 ± 1.12% to 27.45 ± 0.70% (25.47% decrease) as the nitrate concentration decreased. This is similar to the trend of pigment concentration under nitrogen limitation (Figure 2a). Algal cultures containing lower nitrate concentrations (10.83 and 0 mg/L) had a lower protein content (Figure 3a). This was in line with the results observed in *Picocystis salinarum* under nitrogen limitation conditions [17,22]. Compared to nitrogen-rich culture containing 173.25 mg/L nitrate, the total protein showed a decrease of 8.50% and 25.47% in cultures containing 10.83 and 0 mg/L nitrate, respectively. Previous studies also showed a similar decrease in protein content under nitrogen-depleted conditions for *Acutodesmus obliquus* (24.22%) and *Ankistrodesmus* sp. (27.59%) as compared to nitrogen-sufficient conditions [41]. For the nitrogen starvation experiment, sequential removal of nitrate for 3, 6, and 9 days was found to significantly decrease the protein content by 33.31%, 41.55%, and 45.51%, respectively (*p* < 0.05). Both stress strategies resulted in a decrease in protein content. This could be explained by microalgae cultivated under stress conditions possibly degrading the nitrogen-containing macromolecules such as proteins to maintain the nitrogen quota for basic metabolic requirements [17]. Studies have shown that intracellular proteins such as rubisco could be degraded and reutilized as an alternative nitrogen source [22].

When subjected to stress conditions, microalgae can regulate carbon reallocation into different macromolecules for survival. Microalgae grown in nitrogen-rich conditions tend to synthesize proteins, while those grown in nitrogen-limited conditions are apt to accumulate energy-rich compounds, e.g., lipids or carbohydrates [42]. Lipids and carbohydrates are the major cellular storage products under stress conditions. Carbohydrate accumulation is mainly related to the components of cell wall structural and nutritional reserves [43]. Lipids play an important role in the structural composition of the cellular membrane [44]. Reports have suggested that there is a substrate competition relationship between lipid and carbohydrate biosynthesis [19]. 

As shown in Figure 3a, there seemed to be little difference in lipid content of *C. vulgaris* FACHB-1068 under higher nitrate concentrations (>10.83 mg/L). However, the lipid content generally increased as the nitrate concentration decreased. Algal cultures containing lower nitrate concentrations (10.83 and 0 mg/L) had significantly higher lipid contents as compared to the culture containing 173.25 mg/L nitrate (*p* < 0.05, Figure 3a). This was opposite to the trend of protein content and pigment concentration under nitrogen limitation as mentioned above. Our finding was in accordance with results observed in the cultivation of *Scenedesmus* sp. and *P. salinarum* under nitrogen limitation conditions [17,38]. The total lipid content increased from 28.07 ± 0.55% to 33.30 ± 1.58% (a 19% increase) as the nitrate concentration decreased from 173.25 to 0 mg/L. In contrast, the carbohydrate content significantly increased as the nitrate concentration decreased (*p* < 0.05, Figure 3a). The carbohydrate content was negatively correlated to the nitrate levels tested. However, the lipid content was generally increased and the protein content was generally decreased by stress as mentioned above. Therefore, protein content was positively correlated and lipid and carbohydrate contents were negatively correlated to nitrogen levels tested, which was in line with previous studies [17,22,39]. These findings suggested the reallocation of carbon among lipids, proteins, and carbohydrates in response to stress conditions. As seen from Figure 3b, nitrogen starvation had an obvious effect on the biochemical composition of *C. vulgaris* FACHB-1068. Similarly, protein content was also positively correlated and lipid and carbohydrate contents were negatively correlated to nitrogen-starved cultures tested. The lipid and carbohydrate contents increased by 21.11% and 28.19%, respectively, whereas the protein content decreased by 45.51% as the period of nitrogen starvation increased from 0 to 9 days. Notably, it was found that the cell density of 3-day nitrogen starvation was almost the same as that of control (data not shown), indicating that active cell division occurred in 3-day nitrogen starvation. Hence, this might explain the observation that there was a dramatic decrease in protein content under 3-day nitrogen starvation compared to control. When nitrogen starvation occurred, the nitrogen-containing biomolecules such as proteins might be reutilized as the intracellular nitrogen pool to support cell division and other metabolisms. Therefore, the protein content under 3-day nitrogen starvation decreased dramatically compared to control.

Under nitrogen limitation and starvation, lipids and proteins were found to be the major components (~21–38% DCW) in *C. vulgaris* FACHB-1068, followed by carbohydrates (~4–6% DCW). Taken together, for the nitrogen limitation experiment, the present results showed that the DCW, BP, pigment concentration, and protein and lipid contents were all almost unaffected until the nitrate concentration was less than 21.66 mg/L. In particular, as compared to control (173.25 mg/L nitrate), there was a slight increase in the chlorophyll *a* concentration in the culture containing 21.66 mg/L nitrate (Figure 2a). Nitrogen consumption assay showed that the nitrogen became limiting when the starting nitrate concentration was no more than 21.66 mg/L. Besides, the lipid productivity takes into account both the lipid yield in the cells and the biomass produced [45]. Considering the lipid productivity under nitrogen limitation (Table 1), above-mentioned results, and the downstream harvesting cost, the intermediate nitrate concentration of 21.66 mg/L in the growth medium seems to be better for the production of lipid-rich biomass of *C. vulgaris* FACHB-1068.

For the nitrogen starvation experiment, the present results showed that 3-day nitrogen starvation resulted in increased DCW, BP, and lipid content as compared to the control. The chlorophyll *a* concentration was found to slightly decrease under 3-day nitrogen starvation as compared to control. On this basis, considering the highest lipid productivity under 3-day nitrogen starvation (Table 1), 3-day nitrogen starvation is a more suitable option to produce lipid-rich biomass of *C. vulgaris* FACHB-1068. In addition, compared with the results between nitrogen limitation and sequential nitrogen starvation, it can be concluded that the two-step nitrogen starvation strategy seems to be more effective than the nitrogen limitation strategy to produce lipid-rich biomass of *C. vulgaris* FACHB-1068. Thus, the nitrogen starvation strategy was chosen for further study of the effect on fatty acid profile and morphology of *C. vulgaris* FACHB-1068.

### 3.4. Effect of Nitrogen Starvation on Fatty Acid Profile

The lipids extracted from *C. vulgaris* FACHB-1068 under nitrogen starvation were esterified, and the fatty acid composition was determined using gas chromatography. The fatty acid profile is presented in Table 2. The dominant components in *C. vulgaris* FACHB-1068 under nitrogen starvation were saturated fatty acids (SFAs, C16~C20) such as palmitic acid (C16:0), arachidic acid (C20:0), and stearic acid (C18:0) and unsaturated fatty acids (UFAs) such as oleic acid (C18:1). The SFAs accounted for about ~80% of total fatty acids. For SFAs, C16:0 and C18:0 were predominant under all nitrogen starvation conditions, accounting for more than half of the SFAs. The content of C16:0 remained constant, and C14:0 and C18:0 content decreased as the period of nitrogen starvation increased. Noteworthily, a considerable increase was observed in C20:0 content, accompanied by the sizable decrease in C18:1 and C18:2 content in response to stress conditions (Table 2). A similar type of trend in fatty acid conversation was also found in *Nannochloropsis oculate* [36], *P. salinarum* [38], *Nannochloropsis* sp. [46], and *Nannochloropsis oceanica* [47]. With respect to UFAs, the content of C14:1 and C16:1 was unaffected by nitrogen starvation. However, C18:1 content decreased dramatically under 9-day nitrogen starvation compared to control. C18:2 content was not detected under 3-day and 6-day nitrogen starvation.

Nitrogen starvation had a significant effect on the level of SFAs and UFAs in *C. vulgaris* FACHB-1068 (Table 2). Compared to the control (without nitrogen starvation), the SFAs increased significantly, but UFAs decreased significantly under all three conditions of nitrogen starvation (*p* < 0.05). In other words, nitrogen starvation stimulated the conversion of UFAs to SFAs. Similar results were also found in the cultivation of *P. salinarum* under nitrogen limitation, in which algal cells tended to reduce the degree of fatty acid unsaturation under nitrogen limitation [38]. 

The fatty acid profile determines the quality of microalgae-based biodiesel [5]. Reports have shown that *Chlorella* species are suitable candidates for biodiesel production since C16:0 and C18:0 were dominant components in their fatty acid profiles [48]. This was in good agreement with our results showing that C16:0 and C18:0 content accounted for half or more of total fatty acids under nitrogen starvation. The degree of saturation of fatty acids determines the stability and storability of biodiesel produced; therefore, more SFAs make microalgae a better feedstock for biodiesel application. As the SFAs accounted for ~80% of total fatty acids in response to nitrogen stress, the microalgal lipids produced by *C. vulgaris* FACHB-1068 under sequential nitrogen starvation are deemed to be suitable for biodiesel production.

### 3.5. Morphological Changes in Response to Nitrogen Starvation

As mentioned above, under stress conditions, nitrogen-rich compounds such as chlorophyll *a* may be reutilized to support cell growth. Microalgae tend to accumulate high amounts of different energy-rich bio-compounds, e.g., lipids and carbohydrates, to acclimate to nutritional deficiency for survival. The lipids and carbohydrates produced are stored in subcellular structures and are packaged as lipid droplets (lipid body or oil body) and starch granules (starch grain), respectively. Studies have shown that lipids are first produced and packaged in the plastid and then transported to the cytoplasm and form lipid droplets [38]. The lipid droplets form in response to specific cellular needs, and the number of lipid droplets in each cell changes in response to nutritional conditions [49]. Besides, there have been many studies reporting that nitrogen stress can not only boost lipid accumulation, but also lead to chloroplast degradation, photosystem damage, and a final reduction in photosynthetic efficiency [19].

**Figure 4:**
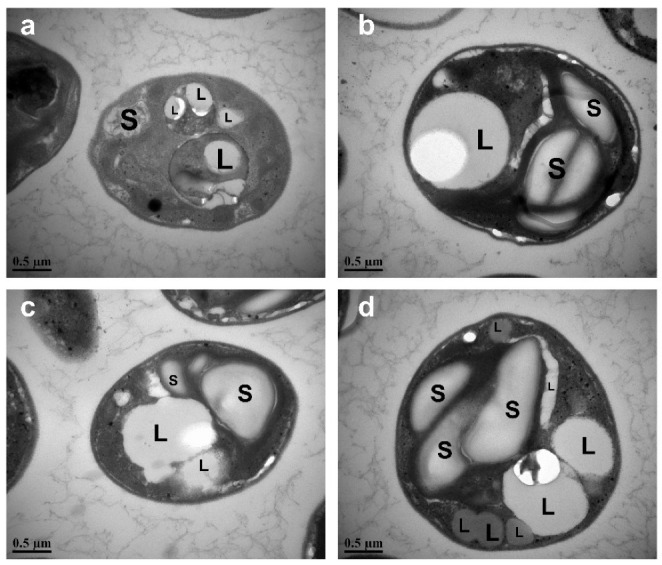
Electron micrographs of *C. vulgaris* FACHB-1068 cultured under different nitrogen starvation conditions: (**a**) control, (**b**) nitrogen starvation for 3 days, (**c**) nitrogen starvation for 6 days, (**d**) nitrogen starvation for 9 days. L, lipid droplet; S, starch granule.

Electron micrographs of *C. vulgaris* FACHB-1068 cells under different periods of nitrogen starvation showed great changes in morphology (Figure 4). Under normal culture conditions without nitrogen stress, the chloroplasts in the cell took up most of the cell volume (Figure 4a). However, under different periods of nitrogen starvation ranging from 3 to 9 days, the chloroplasts underwent an increasing degradation (Figure 4b,c) and became imperceptible. This was in line with the pigment analysis as described above (Figure 2). At the same time, the numbers of both lipid droplets and starch granules increased as the period of nitrogen starvation increased, with the prominent increase in the total size of lipid droplets and starch granules (Figure 4b–d). This observation was also in good agreement with the results of biochemical changes (Figure 3).

The total lipid content in microalgae includes neutral lipids (NLs), glycolipids (GLs), and phospholipids (PLs). NLs, particularly triacylglycerol (TAG), are the most energy-dense storage lipids, while GLs and PLs represent the main structural components of membrane lipids. Detailed information on microalgal lipid class is helpful for a further understanding of lipid accumulation mechanisms under nitrogen stress. Thus, future research may focus on the exploration of lipid class under nitrogen stress to clarify which component of total lipid content contributes greatly to the lipid accumulation in microalgae. Besides, except for nitrate consumption, other aspects such as changes in pH values and phosphate levels also need to be further investigated for a better understanding of the effect of nitrogen stress on cell growth.

## 4. Conclusions

In summary, nitrogen limitation (when nitrate concentration was below 21.66 mg/L) and starvation enhanced the lipid content but generally decreased the biomass productivity, pigment concentration, and protein content in algal cells. In addition, *C. vulgaris* FACHB-1068 is capable of growing in culture containing 87.5% less starting nitrate. As compared to the nitrogen limitation strategy, nitrogen starvation for 3 days is a more suitable approach to increase biomass and lipid production for *C. vulgaris* FACHB-1068. Nitrogen starvation resulted in significant changes in cell morphology, with degraded chloroplasts and increased numbers and total size of lipid droplets and starch granules. Under nitrogen starvation, saturated fatty acids (C16~C20) accounted for the major portion of the total fatty acids (~80%), making our strain a potential feedstock for biodiesel production. Our results may contribute to a better understanding of the biochemical and morphological changes in microalgae under nitrogen stress. Besides, the nitrogen stress strategy used in our work may be useful to maximize the biomass and lipid production of oleaginous microalgae.

## Figures and Tables

**Table 1:** Dry cell weight (DCW), biomass productivity (BP), and lipid productivity of *C. vulgaris* FACHB-1068 under nitrogen limitation and sequential nitrogen starvation.

Parameter	Nitrogen Limitation						Sequential Nitrogen Starvation			
	173.25 mg/L	86.63 mg/L	43.31 mg/L	21.66 mg/L	10.83 mg/L	0 mg/L	Control	3-Day NS	6-Day NS	9-Day NS
DCW (mg/L)	491.67 ± 12.58 ^a^	458.33 ± 11.55 ^b^	438.33 ± 20.21 ^b^	448.33 ± 30.14 ^b^	393.33 ± 17.56 ^c^	403.33 ± 7.64 ^c^	243.33 ± 15.28 ^b^	266.67 ± 2.89 ^a^	226.67 ± 2.89 ^bc^	213.33 ± 15.28 ^c^
BP (mg/L/day)	32.78 ± 0.84 ^a^	30.56 ± 0.77 ^b^	29.22 ± 1.35 ^b^	29.89 ± 2.01 ^b^	26.22 ± 1.17 ^c^	26.89 ± 0.51 ^c^	16.22 ± 1.02 ^b^	17.78 ± 0.19 ^a^	15.11 ± 0.19 ^bc^	14.22 ± 1.02 ^c^
Lipid productivity (mg/L/day)	9.20 ± 0.18 ^a^	8.43 ± 0.53 ^bc^	7.96 ± 0.25 ^c^	8.32 ± 0.03 ^bc^	8.25 ± 0.41 ^c^	8.95 ± 0.43 ^ab^	4.74 ± 0.09 ^a^	5.57 ± 0.02 ^c^	4.93 ± 0.06 ^b^	5.03 ± 0.14 ^b^

Control, algal culture grown in nitrate-sufficient medium for 15 days; 3-Day NS, 6-Day NS, and 9-Day NS, algal culture grown in nitrate-sufficient medium for 12, 6, and 3 days, respectively, followed by nitrogen starvation for 3, 6, and 9 days, respectively. Data are expressed as mean ± SD (*n* = 3). Values with different letters represent significant difference (*p* < 0.05) between treatments.

**Table 2:** Fatty acid composition (% total fatty acids) of *C. vulgaris* FACHB-1068 cultured under different nitrogen starvation conditions.

Fatty Acids	Control	3-Day NS	6-Day NS	9-Day NS
C14:0	4.45 ± 1.12	4.16 ± 0.38	4.06 ± 1.02	3.06 ± 0.20
C14:1	1.13 ± 0.00 ^b^	1.50 ± 0.14 ^a^	1.26 ± 0.03 ^b^	1.22 ± 0.18 ^b^
C16:0	27.64 ± 0.35 ^b^	28.43 ± 0.28 ^a^	27.25 ± 0.44 ^b^	27.20 ± 0.16 ^b^
C16:1	2.98 ± 0.32 ^a^	2.56 ± 0.03 ^b^	2.96 ± 0.09 ^a^	2.45 ± 0.03 ^b^
C18:0	24.42 ± 1.30 ^a^	24.14 ± 0.81 ^a^	23.19 ± 1.35 ^a^	19.06 ± 1.64 ^b^
C18:1 n-9	13.95 ± 1.69 ^b^	16.51 ± 0.24 ^a^	15.29 ± 0.92 ^ab^	4.82 ± 0.67 ^c^
C18:2 n-6	21.76 ± 0.74 ^a^	ND	ND	10.43 ± 0.47 ^b^
C20:0	3.51 ± 0.39 ^d^	22.30 ± 1.24 ^c^	25.58 ± 1.22 ^b^	31.20 ± 1.52 ^a^
C20:5 n-3	ND	0.40 ± 0.05 ^b^	0.42 ± 0.06 ^ab^	0.56 ± 0.13 ^a^
SFAs	60.02 ± 2.38 ^b^	79.03 ± 0.16 ^a^	80.08 ± 0.89 ^a^	80.52 ± 0.29 ^a^
UFAs	39.98 ± 2.38 ^a^	20.97 ± 0.16 ^b^	19.92 ± 0.89 ^b^	19.48 ± 0.29 ^b^
MUFAs	18.07 ± 1.37 ^b^	20.57 ± 0.11 ^a^	19.50 ± 0.89 ^ab^	8.49 ± 0.55 ^c^
PUFAs	21.92 ± 1.01 ^a^	0.40 ± 0.05 ^c^	0.42 ± 0.06 ^c^	10.99 ± 0.37 ^b^

Control, algal culture grown in nitrate-sufficient medium for 15 days; 3-Day NS, 6-Day NS, and 9-Day NS, algal culture grown in nitrate-sufficient medium for 12, 6, and 3 days, respectively, followed by nitrogen starvation for 3, 6, and 9 days, respectively. SFAs, saturated fatty acids. MUFAs, monounsaturated fatty acids. UFAs, unsaturated fatty acids. PUFAs, polyunsaturated fatty acids. Data are expressed as mean ± SD (*n* = 3). ND, not detected. Values with different letters represent significant difference (*p* < 0.05) between treatments.

## Data Availability

The data of this study are available from the corresponding author upon reasonable request.
